# Hepatitis B Core Antigen Impairs the Polarization While Promoting the Production of Inflammatory Cytokines of M2 Macrophages via the TLR2 Pathway

**DOI:** 10.3389/fimmu.2020.00535

**Published:** 2020-03-27

**Authors:** Hongyu Yi, Ye Zhang, Xiaofei Yang, Mengyuan Li, Haifeng Hu, Jie Xiong, Ning Wang, Jingyi Jin, Yusi Zhang, Yun Song, Xian Wang, Lihua Chen, Jianqi Lian

**Affiliations:** ^1^Department of Infectious Diseases, Tangdu Hospital, The Fourth Military Medical University, Xi'an, China; ^2^Department of Immunology, The Fourth Military Medical University, Xi'an, China; ^3^Department of Respiratory and Critical Care, Xijing Hospital, The Fourth Military Medical University, Xi'an, China

**Keywords:** hepatitis B virus, HBV core protein, macrophage polarization, Toll-like receptor 2, inflammatory cytokines

## Abstract

Although several evidences suggesting the vital roles that innate immunity plays in the persistence and elimination of chronic hepatitis B virus (CHB) infection, the exact mechanism is still complicated. Here, we successfully polarized monocytes derived from healthy human peripheral blood mononuclear cells (PBMCs) into M1/M2 macrophages and detected the effects of hepatitis B core antigen (HBcAg) on the polarization and function of macrophages via the Toll-like receptor (TLR) 2 signaling pathway. The results showed that HBcAg had a negligible impact on M1 polarization, while it effectively impaired M2 polarization and promoted the production of pro-inflammatory cytokines such as IL-6 and TNF-α. Additionally, HBcAg treatment increased TLR2 expression on M2 macrophages and TLR2 blockade abolished the effects of HBcAg on the impaired phenotype and pro-inflammatory cytokine productions of M2 macrophages. Signaling pathway analysis revealed that the nuclear factor κB (NF-κB) pathway, the downstream of TLR2, was upregulated upon HBcAg treatment in both M1 and M2 macrophages. Furthermore, a CD8^+^ T-macrophage coculture system implied that compared with PBS stimulation, HBcAg-stimulated M2 macrophages regained their ability to activate CD8^+^ T cells with higher secretion of IFN-γ. Finally, we found impaired expression of M2-related molecules and increased levels of pro-inflammation cytokines in M2 macrophages from CHB patients upon HBcAg stimulation. In conclusion, these results imply a favorable role of HBcAg in the establishment of a pro-inflammatory microenvironment by macrophages, which may suggest a potential therapeutic strategy of HBcAg-induced macrophage activation in CHB infection.

## Introduction

Hepatitis B virus (HBV) infection remains a heavy public health burden that still affects ~250 million people around the world, despite the effective use of HBV vaccination (1). The liver is the main target organ in HBV infection, in which chronic hepatitis can ultimately progress to fibrosis, cirrhosis, and even hepatocellular carcinoma (HCC). However, the eradication of HBV is rarely achieved because of the stable existence of covalently closed circular DNA (cccDNA) in infected hepatocytes (2) and, more importantly, impaired antiviral immune responses, including dysregulated CD8^+^ T cell responses and inactive innate immunity (3). Given the limited efficacy of interferons (IFNs) and nucleoside analog, uncovering the relationship between persistent HBV infection and host immunological tolerance seems quite necessary for future therapeutic approaches.

Macrophages, important performers in mediating adaptive and innate immune responses, are characterized by intricate plasticity as they respond to immune milieu disturbances. The liver has the largest quantity of macrophages among all tissues in the adult human body (4). Liver macrophages have pivotal functions in eliminating pathogenic microbes, initiating or modulating liver inflammatory reactions and sustaining the liver microecology to support body health (5). To date, there are two main branches of liver macrophages: the cells in the largest branch are termed Kupffer cells (KCs), and those in the other are defined as monocyte-derived infiltrating macrophages. Liver diseases such as infection or injury may trigger KC activation, inducing the secretion of pro-inflammatory cytokines to defend pathogens, and may even produce inhibitory cytokines to support liver remodeling and immune tolerance. In contrast, upon pro-inflammatory stimulation, phagocytes derived from the bone marrow or peripheral blood monocytes can pour into the liver promptly, and evidence shows that these infiltrating macrophages can abrogate KC-induced immune tolerance (6, 7). Many studies have revealed the importance of macrophages in mediating immune reactions during HBV infection, especially the inhibitory role of KCs as several studies revealed that depleting liver KCs may prevent HBV infection from becoming chronic (6). However, the antiviral effects of liver monocyte-derived infiltrating macrophages on the process of HBV infection are still unresolved. Such indeterminate conclusions demand further exploration.

Hepatitis B core antigen (HBcAg) is a 183-residue, 21-kDa polypeptide with the first 1–149 residues forming the assembly domain (Cp149), which is dispensable for self-assembly, and the final 34 repeated arginine-rich residues called the C-Terminal domain (CTD), which regulates RNA reverse transcription and nucleic acid binding (8). Self-assembled HBcAg forms the icosahedral viral nucleocapsid that encloses the viral genome and DNA polymerase. Previous studies have suggested that Toll-like receptor (TLR) 2 expressed on macrophages may be involved in the detection of HBcAg (9), and a recent research has provided new insights into the prohibitive role of HBcAg in mediating CD8^+^ T cell exhaustion with an interaction with KCs during HBV infection (10). Nevertheless, a surprising finding identified the role of HBcAg in determining virus elimination in HBV-transfected mice, as the absence of HBcAg resulted in decreased recruitment of infiltrating TNF-α^+^ Ly6C^+^ monocytes than control transfection, which lead to prolonged HBV infection (11). All of these studies indicate the discrepant effects between KCs and monocyte-derived macrophages in response to HBcAg. However, the interplay between monocyte-derived macrophages and HBcAg is still far from being elucidated.

Here, we investigated the underlying influences of HBcAg on macrophage differentiation and functions using monocyte-derived macrophages from healthy individuals or CHB patients. Additionally, as TLR2 expressed on macrophages can recognize the HBcAg, we also explored the interactions between the HBcAg-induced changes in macrophage polarization and the TLR2 signaling pathway as well as the activation of the nuclear factor κB (NF-κB) pathway downstream of TLR2. Finally, we established a coculture system to evaluate the influence of macrophages treated with HBcAg on CD8^+^ T cell activation.

## Materials and Methods

### Chemicals and Reagents

Human recombinant GM-CSF, M-CSF, IFN-γ, and IL-4 were obtained from PeproTech. LPS from *Escherichia Coli* (serotype 055; B5) was obtained from Sigma-Aldrich. Anti-TLR2 antibody (Ab) was purchased from R&D Systems and recombinant HBcAg was purchased from ProSpec. Mouse monoclonal anti-GAPDH and anti-Lamin A/C Abs were purchased from Proteintech. Mouse monoclonal anti-IκBα, rabbit monoclonal anti-phospho-IκBα, anti-NF-κB p65, anti-phospho-NF-κB p65, anti-STAT1, anti-phospho-STAT1, anti-STAT6, and anti-phospho-STAT6 Abs and corresponding HRP-conjugated secondary antibodies were all purchased from Cell Signaling Technology.

### Samples

Nine patients with CHB, and six asymptomatic HBV carriers (AsC) were enrolled in the study (Supplementary Table 1). The standards for diagnosis were determined according to the diagnostic standard of the Chinese National Program for the Prevention and Treatment of Viral Hepatitis. All patients were without evidence of hepatic decompensation, hepatocellular carcinoma (HCC), liver transplant and did not receive antiviral therapy for 6 months before sampling. Patients who were co-infected with human immunodeficiency virus (HIV), or other hepatitis viruses and autoimmune diseases were excluded. The study protocol was approved by the ethics committee of the Tangdu Hospital (Xi'an, China). Buffy coats from 28 healthy donors were provided by the Xijing Hospital Blood Center (Xi'an, China). Informed consent was provided according to the protocols of the Xijing Hospital Blood Center.

### Monocyte Isolation, Polarization, and Stimulation of Human Macrophages

Monocytes were purified from peripheral blood mononuclear cells (PBMCs) obtained from blood buffy coats, followed by an additional step using anti-CD14 magnetic bead (Miltenyi Biotec) according to the manufacturer's protocol. For macrophage differentiation, monocytes were cultured for 6 d in RPMI 1640 medium (HyClone) supplemented with 10% FCS (Corning) and 1% (v/v) penicillin-streptomycin (Solarbio) in the presence of 50 ng/ml GM-CSF (named unpolarized M1 macrophages, M0–M1 MΦ) or 100 ng/ml M-CSF (named unpolarized M2 macrophages, M0–M2 MΦ) in dishes at a density of 1 × 10^6^ cells/ml. Macrophage polarization was obtained by refreshing the culture medium and culturing cells for an additional 24 h in RPMI 1640 medium supplemented with 10% FCS containing 100 ng/ml LPS + 20 ng/ml IFN-γ added to M0–M1 MΦ (named polarized M1 MΦ) or 25 ng/ml IL-4 added to M0–M2 MΦ (named polarized M2 MΦ) as previously described (12). To analyze the effect of HBcAg on macrophage polarization, 10 μg/ml HBcAg or an equal volume of PBS was added simultaneously with LPS/IFN-γ or IL-4 to unpolarized M1 or M2 MΦ and incubated for an additional 24 h for further polarization. For a TLR2 blocking assay, unpolarized M1 and M2 MΦ were pretreated with 0.10 μg/ml anti-TLR2 antibody for 4 h before incubation with LPS/IFN-γ, IL-4, and HBcAg. Freshly isolated monocytes were defined as M0 cells.

### Flow Cytometry

The human monoclonal antibodies (mAbs) used for flow cytometry in this article included FITC-anti-CD3, FITC-anti-CD80, FITC-anti-CD163, FITC-anti-CD8α, FITC-anti-CD14, PE-anti-CD40, APC-anti-IFN-γ (all from BioLegend), BB515-anti-TLR2, and PE-anti-CD206 (all from BD Biosciences). Cells were scrapped from culture plates and incubated with human AB serum to saturate non-specific mAb binding before staining with the indicated fluorophore-labeled mAbs. For intracellular IFN-γ staining, cells were stimulated with Cell Activation Cocktail (with Brefeldin A; BioLegend) for 6 h according to the manufacturer's protocol. All data were collected using Novocyte flow cytometer (ACEA) and analyzed with FlowJo software.

### Immunofluorescent Staining

Immunofluorescence staining was performed as described previously (11). Paraffin-embedded para-carcinoma tissues from HCC patients were stained with rabbit anti-CD68 (Abcam) and mouse anti-HBcAg (Abcam) Abs. After washing with PBS, tissues were incubated with goat anti-rabbit IgG conjugated to Alexa Fluor-488 and anti-mouse IgG conjugated to Cy3. The slides were viewed using an OLYMPUS inverted microscope.

### Reverse Transcription-Real Time Quantitative PCR (RT-qPCR)

After obtaining polarized macrophages, we detected the levels of several polarization-related mRNA transcripts. RT-qPCR was performed as described previously (13). The primes used were as follows: GAPDH: sense, 5′-GAAGGTGAAGGTCGGAGTC-3′, and antisense, 5′-GAAGATGGTGATGGGATTTC-3′; TLR2: sense, 5′-ATCCTCCAATCAGGCTTCTCT-3′, and antisense, 5′-GGACAGGTCAAGGCTTTTTACA-3′; CCR7: sense, 5′-TGAGGTCACGGACGATTACAT-3′, and antisense, 5′-GTAGGCCCACGAAACAAATGAT-3′; CD40: sense, 5′-ACTGAAACGGAATGCCTTCCT-3′, and antisense, 5′-CCTCACTCGTACAGTGCCA-3′; CD80: sense, 5′-AAACTCGCATCTACTGGCAAA-3′, and antisense, 5′-GGTTCTTGTACTCGGGCCATA3′; IL-10: sense, 5′-GGCACCCAGTCTGAGAACAG-3′, and antisense, 5′-ACTCTGCTGAAGGCATCTCG-3′; CD206: sense, 5′-GGGTTGCTATCACTCTCTATGC-3′, and antisense, 5′-TTTCTTGTCTGTTGCCGTAGTT-3′; CD163: sense, 5′-GTCGCTCATCCCGTCAGTCATC-3′, and antisense, 5′-GCCGC-TGTCTCTGTCTTCGC3′; CD86: sense, 5′-TGTCAGTGCTTGCTAACTTCAG-3′, and antisense, 5′-TGGTCATATTGCTCGTAACATCAG-3′.

### Cytokine Detection by Enzyme-Linked Immunosorbent Assay (ELISA)

Levels of TNF-α, IL-6, IL-10, IFN-γ, IL-12, and TGF-β secreted in the culture medium were quantified by commercial ELISA kits according to the manufacturer's instructions (Westang, Shanghai, China).

### Western Blot Analysis

After being stimulated with GM-CSF or M-CSF for 6 d, unpolarized M0–M1 and M0–M2 MΦ were incubated with LPS/IFN-γ or IL-4, respectively, for further differentiation. After an additional 24 h incubation, the polarized M1 or M2 MΦ were stimulated with 10 μg/ml HBcAg (ProSpec) or PBS for another 30 min. The cells were washed twice with PBS and lysed in ice-cold lysis buffer [50 mM Tris-HCl (pH 7.4), 150 mM NaCl, 2 mM NaN_3_, 5 mM EDTA, 0.1% SDS, and 2% Triton X-100] containing a mixture of protease inhibitors and PhosSTOP Phosphatase Inhibitor (Roche Molecular Biochemicals) for 30 min at 4°C. The lysates were obtained after a centrifugation at 12,000 × g to remove nuclei. Equal volumes and amounts of protein were electrophoresed and separated by a SDS-PAGE, and transferred to a nitrocellulose membrane (Millipore). After the membrane was blocked using TBST containing 5% skim milk and, then incubated with primary Abs at 4°C overnight followed by incubation with an HRP-conjugated secondary Ab for 1 h at 37°C. The membrane was visualized using ECL (Millipore).

### CD8^+^ T-Macrophage Coculture System

In total, 1 × 10^5^ freshly purified autologous CD8^+^ T cells obtained from PBMCs were cocultured alone or with 5 × 10^4^ polarized M1 or M2 macrophages (stimulated with HBcAg or not) in the presence of 3 μg/ml anti-CD3/anti-CD28 Abs (BioLegend) for 3 d. Intracellular IFN-γ staining was performed to analyze the amount of IFN-γ-producing CD8^+^ T cells. Supernatants were collected to assess the cytokine concentrations by ELISA.

### Statistical Analysis

All data are presented as the result of three or four independent experiments and expressed as the mean ± SEM. GraphPad Prism 7 (GraphPad Software, San Diego, CA, USA) was used to evaluate these data. A paired Student's *t*-test was applied to determine the significant differences between groups. *P* < 0.05 were defined as statistically significant.

## Results

### Comparison of Human Peripheral Monocyte-Derived M1 and M2 Macrophage Polarization Profiles

**Figure 1:**
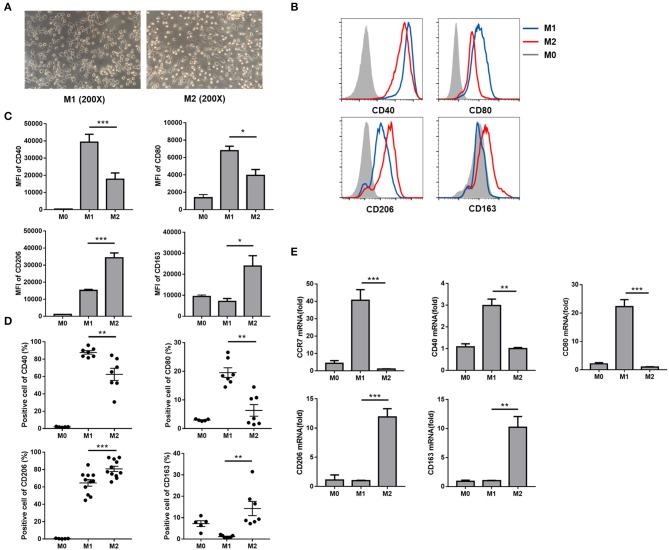
Comparison of human peripheral monocyte-derived M1 and M2 macrophage polarization profiles. **(A)** After M1 and M2 MΦ were eventually polarized upon an additional 24 h incubation with LPS/IFN-γ or IL-4, the morphology of M1 and M2 MΦ were observed using light microscope (original magnification, × 200). **(B)** Representative flow cytometric graphs showing CD40, CD80, CD206, and CD163 expression levels in M1 and M2 MΦ. Cumulative results calculating MFI **(C)** and positive cells **(D)** of CD40, CD80, CD206, and CD163 expression levels in freshly isolated monocytes (M0), M1, and M2 MΦ. **(E)** CCR7, CD40, CD80 (upper), CD206, and CD163 (lower) mRNA levels in M0, M1, and M2 MΦ were analyzed by RT-qPCR. Experiments in **(B–E)** were repeated at least two times. Data were analyzed using a paired Student's *t*-test. Results are shown as the mean ± SEM (**B–D**, *n* = 6–11; **E**, *n* = 3). *p < 0.05, **p < 0.01, ***p < 0.001.

To investigate the effects of HBcAg on macrophage polarization, we polarized human peripheral monocyte-derived macrophages into the M1 or M2 phenotype by stimulating monocytes with GM-CSF or M-CSF for 5–6 d and administering an additional dispose of LPS/IFN-γ or IL-4, respectively, for 24 h *in vitro* (14). Macrophage morphological analysis found that M1 macrophages showed a round shape as a result of GM-CSF treatment and additional LPS/IFN-γ stimulation, while M2 macrophages mainly exhibited a spindly and elongated morphology in the presence of M-CSF and IL-4 (Figure 1A). Next, we investigated the expression of phenotypic markers of these completely polarized MΦ. As previous studies revealed (15), we found that M1 MΦ expressed more costimulatory molecules such as CD40 and CD80 than M2 MΦ, while macrophage mannose receptor (MMR), namely CD206, and CD163 were both upregulated in M2 MΦ compared with M1 MΦ (Figures 1B–D). Analysis of the mRNA expression of polarization-related genes revealed the same results with additional confirmation that the M1-related marker CCR7 was highly expressed on LPS/IFN-γ treated polarized M1 MΦ (Figure 1E). Taken together, we successfully established a polarized macrophage model.

### HBcAg Selectively Impaired M2 Macrophage Phenotype and Retrieved the Production of Inflammatory Cytokines

**Figure 2:**
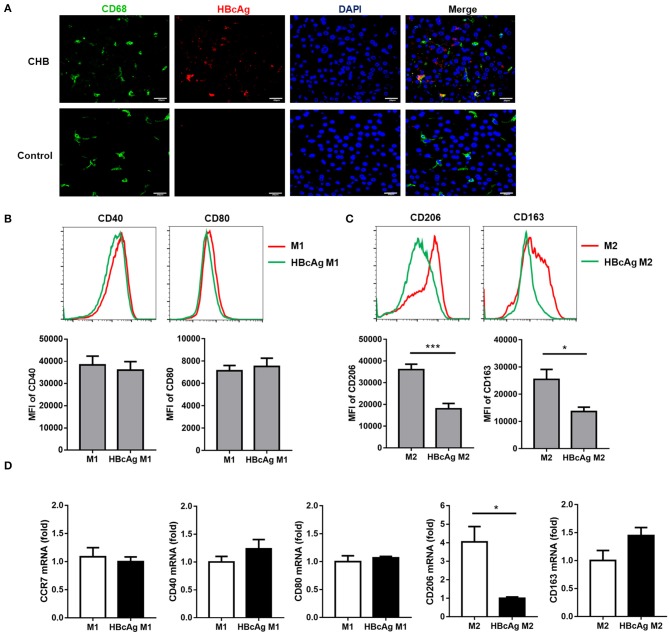
HBcAg selectively inhibited M2 macrophage phenotype. **(A)** The co-localization between HBcAg and CD68 was detected by immunofluorescence staining. Red and green represent HBcAg and CD68, respectively, blue represents nuclei and yellow represents co-localization. **(B–D)** 10 μg/ml HBcAg or an equal volume of PBS was added simultaneously with LPS/IFN-γ or IL-4 to unpolarized M1 or M2 MΦ and incubated for an additional 24 h for further polarization. **(B)** Representative flow cytometric graphs (upper) and cumulative results (lower) showing CD40 and CD80 expression levels in M1 MΦ stimulated with either PBS or HBcAg. **(C)** Representative flow cytometric graphs (upper) and cumulative results (lower) showing CD206 and CD163 expression levels in M2 MΦ stimulated with either PBS or HBcAg. **(D)** CCR7, CD40, and CD80 mRNA levels in M1 MΦ or CD206 and CD163 mRNA levels in M2 MΦ stimulated with either PBS or HBcAg were analyzed by RT-qPCR. Experiments in **(A–D)** were repeated at least two times. Data were analyzed using a paired Student's *t*-test. Results are shown as the mean ± SEM (**B–C**, *n* = 8; **D**, *n* = 3). *p < 0.05, ***p < 0.001.

As a previous study found that HBcAg could induce cytokine productions in human monocytic cell line (THP-1) (9), we hypothesized that HBcAg might play an important role in interfering with the function and phenotype of macrophages. Although the HBcAg is thought to normally envelop inside hepatocytes, several studies have already demonstrated that HBcAg may be released from HBV infected hepatocytes and then trigger macrophage activation (16). Here, we found the colocalization of HBcAg (red) and CD68 (green) using immunofluorescence staining of liver tissues from HBV-infected and HBV-negative HCC patients, which indicated that HBcAg may directly interact with macrophages (Figure 2A).

To further discuss the effect of HBcAg on macrophages, we added 10 μg/ml HBcAg or an equal volume of PBS simultaneously with LPS/IFN-γ or IL-4 to unpolarized M1 or M2 MΦ, respectively, and incubated for further polarization, followed by flow cytometry and RT-qPCR analyses. The supernatants of the cell cultures were collected and then analyzed using ELISA for IL-10, IL-6, TNF-α, IL-12, and TGF-β concentrations. To our surprise, the levels of the M2-related markers CD206 and CD163 on monocyte-derived M2 macrophages were significantly reduced by HBcAg treatment, although the mRNA expression of CD163 showed no statistical significance between the PBS and HBcAg groups (Figures 2C,D). However, compared with PBS treatment, the presence of HBcAg did not seem to affect the expression of CD40, CD80, or CCR7 on monocyte-derived M1 macrophages (Figures 2B,D). We further assessed if the HBcAg treatment can polarize M2 macrophages toward M1. As shown in Supplementary Figure 3A, HBcAg-treated M2 macrophages did not alter the level of CD40 or CD80 compared with control group.

**Figure 3:**
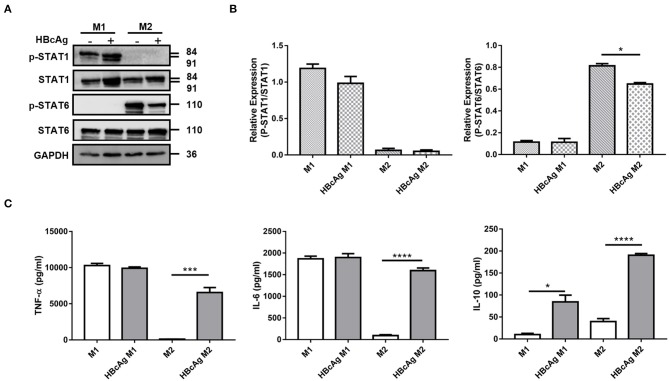
HBcAg retrieved the production of inflammatory cytokines by M2 macrophages and inhibited M2 phenotype correlated with STAT6 pathway. **(A,B)** After being stimulated with GM-CSF or M-CSF for 6 d, M0–M1 and M0–M2 unpolarized MΦ were incubated with LPS/IFN-γ or IL-4 for further differentiation. After another 24 h incubation, polarized M1 or M2 MΦ were stimulated with 10 μg/ml HBcAg or an equal volume of PBS for another 30 min. The protein levels of STAT1, p-STAT1, STAT6, and p-STAT6 were analyzed by Western blot. **(A)** Representative figures and **(B)** cumulative densitometry quantification for p-STAT1 and p-STAT6 expression normalized to STAT1 and STAT6, respectively. **(C)** Supernatants of polarized M1 and M2 MΦ stimulated with HBcAg or PBS were collected to measure TNF-α, IL-6, and IL-10 productions by ELISA. Experiments in **(A,B)** were repeated at least three times. Data were analyzed using a paired Student's *t*-test. Results are shown as the mean ± SEM (**A,B**, *n* = 3; **C**, *n* = 9–11). *p < 0.05, ***p < 0.001, ****p < 0.0001.

It has been reported that the STAT pathway is involved in macrophages polarization, and Genomatix analysis revealed STAT1 and STAT6 as the central hubs of M1 and M2 macrophages polarization, respectively (17). To clarify the mechanism by which HBcAg regulates macrophage polarization, we tested the activation of the abovementioned STAT signaling pathways. As shown in Figures 3A,B, the phosphorylation of STAT6 in M2 macrophages was significantly decreased after stimulation with HBcAg. However, although the total expression of STAT1 was upregulated after HBcAg treatment in M1 macrophages, the ratio of p-STAT1/STAT1 was consistent between PBS and HBcAg-treated M1 macrophages (Figures 3A,B).

Next, we evaluated the cytokine secretion of M1 and M2 polarized macrophages stimulated with HBcAg or PBS. We observed that GM-CSF and LPS/IFN-γ-induced M1 macrophages produced higher levels of pro-inflammatory cytokines such as IL-6 and TNF-α than IL-4-induced M2 macrophages, while M2 macrophages had higher the expression of the inhibitory cytokine IL-10 than M1 macrophages (Figure 3C; Supplementary Figure 2). Furthermore, when we assessed the effect of HBcAg, we found that M2 macrophages exhibited a restored ability to secrete the pro-inflammatory cytokines IL-6 and TNF-α after activation by HBcAg (Figure 3C). However, consistent with previous results for the phenotypic changes in M1 macrophages, HBcAg did not alter the level of IL-6 or TNF-α produced by M1 macrophages (Figure 3C). Interestingly, both M1 and M2 macrophages upregulated the expression level of IL-10 after HBcAg stimulation (Figure 3C; Supplementary Figure 2). It has been reported that IL-6, LPS, and other inflammatory factors can promote IL-10 production, which initiates the normal negative feedback signaling pathway (18). However, the low secretion level of IL-10 did not seem adequate to abolish the proinflammatory cytokine expression of HBcAg-treated M2 macrophages (Figure 3C).

M2 macrophages can be further divided into three subtypes including M2a (induced by IL-4/IL-13 and defined as CD206^hi^), M2b (induced by immune complexes or FcγR/TLR and defined as IL-10^hi^ CD86^hi^ IL-12^low^) and M2c (induced by IL-10 or glucocorticoids and defined as IL-10^hi^ TGF-β^hi^) (19). The polarized macrophage model established in this paper was the M2a subtype. Although HBcAg increased the expression of IL-10 in M2 macrophages, the TGF-β expression was decreased (Supplementary Figure 3D). In addition, HBcAg treatment promoted IL-12 expression in M2 macrophages but did not change the level of CD86 (Supplementary Figures 3B,C). Therefore, these data shows that HBcAg may impair the phenotype of M2a but cannot help M2 macrophages polarize toward M2b or M2c.

Taken together, these results indicate that HBcAg may selectively inhibit M2 macrophage polarization and facilitate the production of inflammatory factors by M2 macrophages.

### TLR2 Blockade Abolished the Impaired M2 Polarization Induced by HBcAg

**Figure 4:**
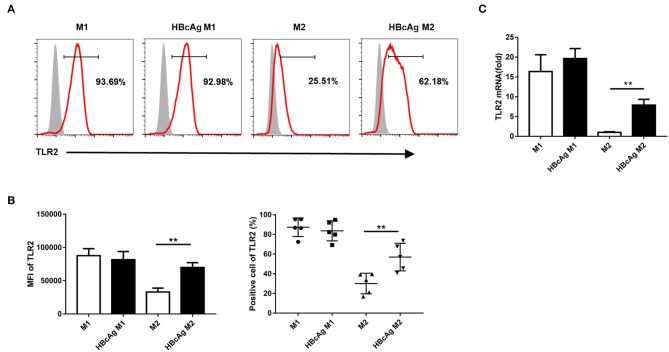
TLR2 expression in M2 macrophages was upregulated via HBcAg stimulation. **(A–C)** 10 μg/ml HBcAg or an equal volume of PBS was added simultaneously with LPS/IFN-γ or IL-4 to unpolarized M1 or M2 MΦ and incubated for an additional 24 h for further polarization. **(A)** Representative flow cytometric graphs and **(B)** cumulative results calculating MFI and positive cells showing TLR2 expression in M1 and M2 MΦ stimulated with either PBS or HBcAg. **(C)** TLR2 mRNA level in M1 and M2 MΦ stimulated with either PBS or HBcAg was analyzed by RT-qPCR. Experiments in **(A–C)** were repeated at least two times. Data were analyzed using a paired Student's *t*-test. Results are shown as the mean ± SEM (**A,B**, *n* = 5; **C**, *n* = 3). **p < 0.01.

**Figure 5:**
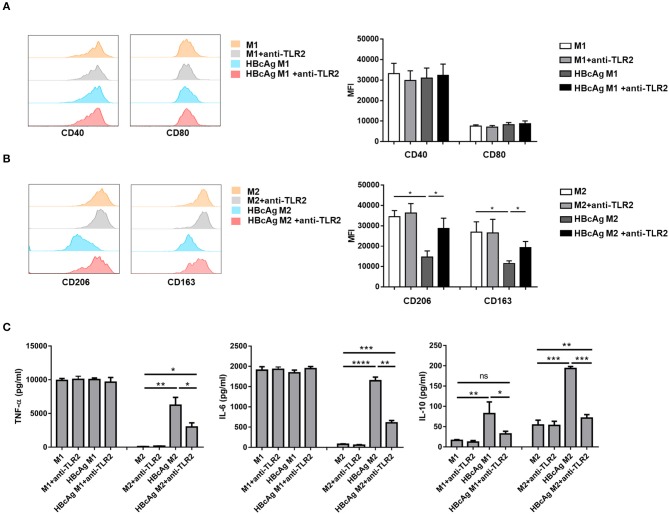
TLR2 blockade abolished the impaired M2 polarization induced by HBcAg. **(A–C)** For TLR2 blocking assay, unpolarized M1 and M2 MΦ were pretreated with 0.10 μg/ml anti-TLR2 for 4 h before incubated with LPS/IFN-γ, IL-4, HBcAg, or PBS. **(A)** Representative flow cytometric graphs (left) and cumulative MFI (right) showing CD40 and CD80 expression levels in M1 MΦ. **(B)** Representative flow cytometric graphs (left) and cumulative MFI (right) showing CD206 and CD163 expression levels in M2 MΦ. **(C)** Supernatants of M1 and M2 MΦ stimulated with HBcAg, PBS, and anti-TLR2 were collected to measure TNF-α, IL-6, and IL-10 productions by ELISA. Experiments in **(A,B)** were repeated at least two times. Data were analyzed using a paired Student's *t*-test. Results are shown as the mean ± SEM (**A–C**, *n* = 5). *p < 0.05, **p < 0.01, ***p < 0.001, ****p < 0.0001.

Because TLRs expressed on macrophages contribute to the detection of pathogens (20) and it has been reported that TLR2 is involved in the recognition of HBcAg by macrophages (9, 10), we further examined whether the inhibition of M2 polarization induced by HBcAg occurs in a TLR2-dependent manner. We found that in comparison to that of PBS-treated M2 macrophages, the expression level of TLR2 of M1 macrophages treated with HBcAg was aproximately 2-fold higher (Figures 4A,B). Results for the mRNA level of TLR2 also confirmed this finding (Figure 4C). However, the presence of HBcAg did not affect the TLR2 expression of M1 macrophages which was already at a relatively high level (Figures 4A–C). Having confirmed the interaction between HBcAg and TLR2 on macrophages, we next blocked TLR2 to further study its relevance. We pretreated unpolarized M1 or M2 macrophages with anti-TLR2 Ab for 4 h and subsequently treated the cells with LPS/IFN-γ, IL-4, and HBcAg. As shown in Figure 5B, TLR2 blockade restored the impaired expression of CD206 and CD163 on HBcAg-treated M2 macrophages. In addition, the blockade of TLR2 did not affect the basic expression of polarization-related markers on M1 and M2 macrophages (M1 vs. M1+anti-TLR2 and M2 vs. M2+anti-TLR2) (Figures 5A,B). Additionally, we detected the production of cytokines. As expected, anti-TLR2 Ab treatment partially abolished the cytokine production of M2 macrophages induced by HBcAg (Figure 5C). Taken together, these results indicated that HBcAg did interfered with the TLR2 signaling pathway in the process of attenuating M2 differentiation under our experimental conditions.

### HBcAg-Responsive M2 Macrophages Exhibited the NF-κB Pathway Activation and Mediated CD8^+^ T Cell Activation

After ligand binding, TLRs interact with intracellular adapters immediately and then activate downstream signaling molecules, such as NF-κB and interferon regulatory factor 1/7 (IRF1/7), which can trigger the production of IFN-α/β or proinflammatory cytokines (21). Moreover, our results revealed the interaction between HBcAg and TLR2 involved in inducing the pro-inflammatory cytokine activation. To further clarify the mechanism by which HBcAg regulates cytokine production, we next investigated the NF-κB pathway activation.

**Figure 6:**
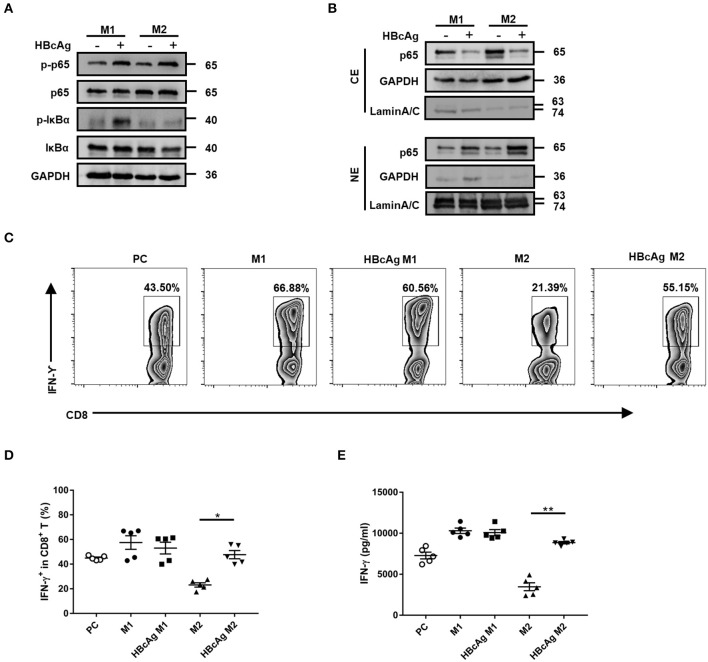
HBcAg-responsive M2 macrophages exhibited the NF-κB pathway activation and mediated CD8^+^ T cell activation. **(A,B)** After being stimulated with GM-CSF or M-CSF for 6 d, M0–M1 and M0–M2 unpolarized MΦ were incubated with LPS/IFN-γ or IL-4 further differentiation. After another 24 h incubation, polarized M1 or M2 MΦ were stimulated with 10 μg/ml HBcAg or an equal volume of PBS for another 30 min. The protein levels of NF-κB p65, p-NF-κB p65, IκBα, and p-IκBα were analyzed by Western blot. Representative figures of total protein levels **(A)** and separated cytosol and nuclear protein levels **(B)** were shown. **(C–E)** 1 × 10^5^ freshly purified autologous CD8^+^ T cells were coculture alone or with 5 × 10^4^ polarized M1 or M2 macrophages (stimulated with HBcAg or not) in the presence of 3 μg/ml anti-CD3/anti-CD28 Abs for 3 d. **(C)** Representative flow cytometric graphs and **(D)** cumulative results showing the amount of IFN-γ-producing CD8^+^ T cells. **(E)** Supernatants of cocultured CD8^+^ T cells were collected to measure IFN-γ production by ELISA. Experiments in **(A,B)** were repeated at least three times. Data were analyzed using a paired Student's *t*-test. Results are shown as the mean ± SEM (**C–E**, *n* = 5). *p < 0.05, **p < 0.01.

Polarized M1 or M2 macrophages were treated with HBcAg for 30 min and the phosphorylation of IκBα and NF-κB p65 was assessed by Western blot. As shown in Figures 6A,B, treatment with HBcAg interfered with the NF-κB pathway, as the phosphorylation of IκBα and NF-κB p65 both increased in HBcAg-treated M2 macrophages compared with PBS-treated M2 macrophages. Interestingly, the phosphorylation of IκBα and NF-κB p65 was also upregulated in HBcAg-treated M1 macrophages compared with PBS-treated M1 macrophages, even though HBcAg did not affect the production of cytokines by M1 macrophages (Figure 6A). This result was further confirmed by subcellular fractionation analysis. We found that the expression of NF-κB p65 in cytoplasmic extracts of M1 or M2 macrophages was reduced, while it was increased in nuclear extracts after HBcAg stimulation (Figure 6B). Taken together, the increased phosphorylation of IκBα and NF-κB p65 and elevated nuclear translocation of NF-κB p65 both indicated that HBcAg could induce the NF-κB pathway activation.

CD8^+^ T cells play a major role in HBV eradication (22, 23), so we wondered whether HBcAg-responsive M2 macrophages exert an influence on CD8^+^ T cells. We employed a coculture assay to investigate the capacity of polarized macrophages to stimulate the IFN-γ production by autologous CD8^+^ T cells activated with anti-CD3/anti-CD28 Abs. Using flow cytometry, we found that the basal number of IFN-γ^+^ CD8^+^ T cells was upregulated in the CD8^+^ T-M1 macrophage coculture system, but reduced in the CD8^+^ T-M2 macrophage coculture system compared with the system containing anti-CD3/anti-CD28 Abs-stimulated CD8^+^ T cells only (used as a positive control, PC) (Figures 6C,D). As expected, pretreatment with HBcAg significantly promoted the ability of M2 macrophages to enhance IFN-γ expression in CD8^+^ T cells. However, there was no difference in IFN-γ production between CD8^+^ T cells primed by HBcAg-treated M1 macrophages and those cells primed by M1 macrophages (Figures 6C,D). Moreover, analysis of IFN-γ secretion in the supernatant by cocultured CD8^+^ T cells also produced the same results (Figure 6E). Therefore, our results indicated that HBcAg pretreatment could in part diminish the suppressive property of M2 macrophages and promote CD8^+^ T cell activation.

### Monocyte-Derived Macrophages of Patients With Chronic HBV Infection Presented a Diminished M2 Subtype Frequency and Enhanced Pro-inflammatory Property in Response to HBcAg Stimulation

**Figure 7:**
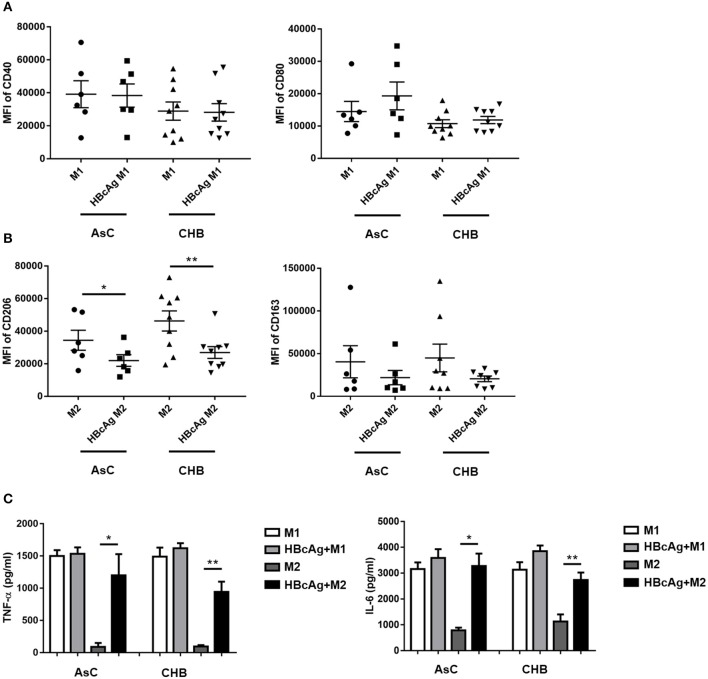
Monocyte-derived macrophages of patients with HBV infection presented a diminished M2 subtype and enhanced pro-inflammatory property under HBcAg stimulation. **(A–C)** 10 μg/ml HBcAg or an equal volume of PBS was added simultaneously with LPS/IFN-γ or IL-4 to unpolarized M1 or M2 MΦ from HBV-infected patients and incubated for an additional 24 h for further polarization. **(A)** Cumulative results showing CD40 and CD80 expression levels in M1 MΦ stimulated with either PBS or HBcAg from asymptomatic HBV carriers (AsC, *n* = 6) or patients infected with chronic hepatitis B (CHB, *n* = 9). **(B)** Cumulative results showing CD206 and CD163 expression levels in M2 MΦ stimulated with either PBS or HBcAg from asymptomatic HBV carriers (AsC, *n* = 6) or patients infected with chronic hepatitis B (CHB, *n* = 9). **(C)** Supernatants of polarized M1 and M2 MΦ from asymptomatic HBV carriers (AsC, *n* = 5) or patients with chronic hepatitis B (CHB, *n* = 5) were collected to measure TNF-α and IL-6 productions by ELISA after stimulated with HBcAg or PBS. Data were analyzed using a paired Student's *t*-test. Results are shown as the mean ± SEM. *p < 0.05, **p < 0.01.

To explore whether our findings obtained using monocyte-derived macrophages from healthy individuals agree with results for macrophages in the context of HBV infection, we examined macrophages derived from patients with AsC or CHB (Supplementary Table 1) to investigate the effects of HBcAg on macrophage differentiation and function. In accord with the results for healthy individuals, the mean fluorescence intensity (MFI) of CD206 on M2 macrophages was successfully attenuated in HBcAg-treated cells from AsC or CHB patients while, the level of CD163 expressed on M2 macrophages only showed a slight decrease upon HBcAg stimulation (Figures 7A,B; Supplementary Figures 1A,B). Besides, HBcAg stimulation also facilitated IL-6 and TNF-α production in the HBcAg-responsive M2 macrophages of AsC or CHB patients (Figure 7C). In conclusion, both healthy individual and CHB patient-derived M2 macrophages exhibited an impaired phenotype and intensified proinflammatory properties upon HBcAg stimulation.

## Discussion

In the current study, using monocyte-derived macrophages obtained from healthy individuals or CHB patients, we determined the effects of HBcAg on macrophage polarization and further assessed the involvement of the TLR2 signaling pathway. The results indicated that HBcAg treatment impaired the M2 phenotype of M2 macrophages and promoted proinflammatory capacity of M2 macrophages, as shown by the increased expression of IL-6 and TNF-α. However, HBcAg may not help M2 macrophages acquire the phenotype of M2b, M2c, or even M1 macrophages. Moreover, CD8^+^ T-macrophage coculture analysis also suggested that M2 macrophages can enhance CD8^+^ T cell function upon pretreatment with HBcAg. Further investigation revealed that HBcAg stimulation triggered TLR2 expression on M2 macrophages and that TLR2 blockade could partially recover the inhibited expression of M2-related molecules and abrogate cytokine production by M2 macrophages. Activation of the downstream NF-κB signaling in HBcAg-treated M1 or M2 macrophages also confirmed the underlying interaction between the HBcAg and TLR2.

Growing evidence reveals the indispensable role for innate immunity in antiviral response. Upon interaction, pattern-recognition receptors (PRRs), such as TLRs, RIG-I-like receptors, and NOD-like receptors (NLRs), recognize specific pathogens and then induce an inflammatory response through the production of proinflammatory cytokines and chemokines, which can directly eradicate the virus or eventually activate the adaptive immune response (24). However, the role of the innate immune system is quite ambivalent in HBV infection. Many studies have confirmed that innate immune responses may be hampered during HBV infection (25), as a recent research found that liver specimens from chronic HBV infected patients did not exhibit expression of more IFN-stimulated genes (ISGs) than those from patients without HBV infection (26). Nevertheless, using a coculture system, researches suggested that although HBV escapes IFN-induced innate immunity mediated by hepatocytes, the virus can be recognized by liver macrophages, causing the release of inflammatory cytokines including IL-6, TNF-α, and IL-1β (27), by which liver macrophages can recruit and activate NK cells, T cells, or B cells and exert collective antiviral effects (28). Such contradictory results indicate the intricate character of the immune response during HBV infection. Macrophages, serving as important innate immunocytes, are involved in almost every aspect of biological responses ranging from tissue development, healing, and homeostasis to antipathogen roles. There are already several studies focusing on the role of macrophages in HBV infection; however, the interplay between the viral antigens and macrophages is poorly demonstrated.

HBcAg, acting as an important stimulus in a host of stages of HBV life cycle, possesses the strong immunogenicity. It is said that HBcAg-specific CD8^+^ T cells of CHB patients may not totally exhausted, therefore can help eliminate HBV infection (29, 30). Besides, Cooper et al. found the core protein of HBV could upregulate the activation of NF-κB and promote inflammatory cytokine release in THP-1 macrophages *in vitro*. Similarly, a surprising finding identified the role for HBcAg in determining virus elimination in HBV-transfected mice (31), as HBV clearance in mice injected with an HBcAg-null plasmid was delayed compared to a wild-type HBV plasmid (11). These studies demonstrate that HBcAg is required for triggering immune responses *in vivo* and *in vitro*. Here, we tested the changes in macrophage polarization in the presence of HBcAg using primary cells isolated from healthy or HBV-infected individuals, which seemed to successfully mimic the cellular properties of humans' better than cell lines and murine cells. We found that HBcAg impaired the M2 phenotype and promoted the production of proinflammatory cytokines such as IL-6 and TNF-α by M2 macrophages from healthy individuals or CHB patients. In contrast, Tian et al. recently showed that HBcAg might facilitate inhibitory IL-10 release from liver-resident KCs and eventually contribute to HBV persistence by inducing CD8^+^ T cell exhaustion in HBV-carrier mice (10). These results are consistent with those of another study that confirmed the inhibitory effects of HBcAg mediated by stimulating IL-10 secretion in T cells and monocytes (32). Our results indeed suggested that HBcAg could induce M1/M2 macrophages to produce the inhibitory cytokine IL-10. However, such a meager IL-10 level seems insufficient to restrain HBcAg-induced macrophage activation because our *in vitro* coculture assay with polarized macrophages/CD8^+^ T cells showed that HBcAg-responsive M2 macrophages prompted IFN-γ production by CD8^+^ T cells.

These controversial opinions on the role of HBcAg in interactions with macrophages might be due to the complicated subtypes and functions of macrophages. The liver harbors two branches of macrophages: the larger one is resident KCs and the other is infiltrating monocyte-derived macrophages. Acting as scavenger cells that sense and phagocytose bacteria and cell debris and present particle-associated antigens, KCs have been considered to contribute greatly to establishing immune tolerance (33, 34), which can protect the liver from experiencing abnormal inflammatory damage and maintain homeostasis. During HBV infection, KCs are thought to exert inhibitory effects contributing to the persistence of HBV infection (6, 35). However, liver inflammation can abolish the immune tolerance induced by KCs and promote inflamed monocyte-derived macrophage infiltration (7), which may be observed in HBV-infected patients (36, 37). A recent research indicated that mice that obliterated HBV harbored a smaller number of IL-10-secreting KCs and a higher number of TNF-α-secreting Ly6C^+^ monocytes than mice in which HBV persisted, which further expounds on the exact roles of KCs and infiltrating monocytes/macrophages in HBV infection (11). Hence, different cells employed in the abovementioned studies may account for the discrepant results. The effect of HBcAg on macrophage responses remains to be investigated.

TLRs, especially TLR2, are thought to play vital roles in recognizing virions and mediating the antiviral immune response during HBV infection (38, 39). Although an *in vitro* assay demonstrated that HBcAg could be recognized by TLR2, resulting in macrophage activation, it is questionable whether core proteins are normally enveloped in virions within HBV-infected hepatocytes and can barely be detected in the peripheral blood, which should prevent HBcAg from being detected by TLR2 expressed on liver macrophages (40). Nonetheless, once the death of HBV-infected hepatocytes occurs, HBc proteins released from these cells may be subsequently captured by liver macrophages via TLR2 detection (16). Consistent with previous studies, we found the colocalization between HBcAg and macrophages in liver tissues from HBV-infected HCC patients. Additionally, in the present study, we also discovered that HBcAg treatment increased the level of TLR2 expression on M2 macrophages, which correlated with the inhibited phenotype and recovered proinflammatory capacity of M2 macrophages upon HBcAg stimulation. These findings extended the relationship between TLR2 and HBcAg-induced macrophage activation. Previous studies have indicated the inhibitory effects of hepatitis B surface antigen (HBsAg), e antigen (HBeAg), and HBV virions on the TLR-induced antiviral response which contributes to HBV persistence (41–43). Additionally, entecavir (ETV) treatment is thought to repair the expression and function of TLR2 in chronically woodchuck hepatitis virus (WHV)-infected woodchucks (44), which was confirmed by a report showing that pre-activation of TLR2 can restore the CD8^+^ T cell response and promote HBV clearance (45).

The activation of the TLR2 signaling pathway is involved in the recruitment of TIRAP and MyD88 and the subsequent activation of the downstream NF-κB pathway, which results in the production of proinflammatory cytokines (20). We detected the role of HBcAg in the regulation of the NF-κB pathway in M2 macrophages. In this study, we found that HBcAg enhanced the activation of NF-κB in M2 macrophages by upregulating the phosphorylation of NF-κB p65 and IκBα. Interestingly, although HBcAg did not interfere with the phenotype or cytokine production of M1 macrophages, it still increased NF-κB activation in M1 macrophages, which might be attributed to TLR2 recognition.

The mechanisms by which HBcAg regulates the polarization and function of macrophages remain unclear. Recently, researchers have found that TRIM29, known as the E3 ubiquitin ligase, may modulate the activation of alveolar macrophages (AMs) in response to viral and bacterial infections via the degradation of NEMO, therefore limits the production of IFN-α/β as well as proinflammatory cytokines of AMs (46). Whether HBcAg can regulate macrophage activation through TRIM29 merits further exploration.

In conclusion, the findings of the present study demonstrated that HBcAg can selectively impair the M2 phenotype and promote the production of inflammatory cytokines by M2 macrophages via TLR2 pathway activation. In addition, HBcAg-responsive M2 macrophages enhanced the activation of CD8^+^ T cells. Thus, these results unveil the favorable effect of HBcAg on the immune response and, more importantly, suggest a potential therapeutic application for TLR2 and HBcAg-induced macrophage activation in CHB infection.

## Data Availability Statement

The datasets generated for this study are available on request to the corresponding author.

## Ethics Statement

The studies involving human participants were reviewed and approved by the ethics committee of Tangdu Hospital (Xi'an, China) and the ethics committee of Xijing Hospital (Xi'an, China). The patients/participants provided their written informed consent to participate in this study.

## Author Contributions

LC and JL designed and instructed the study. HY, ML, HH, JX, NW, JJ, and XW preformed the experiments and analyzed the data. HY, LC, and JL wrote the manuscript. XY, YeZ, YuZ, JX, NW, and YS provided materials and technical supports and offered the constructive proposals. YeZ, LC, and JL reviewed the final manuscript.

### Conflict of Interest

The authors declare that the research was conducted in the absence of any commercial or financial relationships that could be construed as a potential conflict of interest.
