# Supplementary material for: Hepatitis B Core Antigen Impairs the Polarization While Promoting the Production of Inflammatory Cytokines of M2 Macrophages via the TLR2 Pathway
Source: Front Immunol. 2020 Mar 27;11:535. doi: 10.3389/fimmu.2020.00535 (PMC7118225; doi:10.3389/fimmu.2020.00535)
Supplement: Supplementary file 1 [file Table_1.docx]

**Supplementary Table 1**. Clinical characteristic of the study subjects

| Group | AsC | CHB |
| --- | --- | --- |
| Number | 6 | 9 |
| Age (years) * | 30 (24, 44) | 32 (20, 47) |
| Gender  (male/female) | 3/3 | 8/1 |
| ALT (U/L) * | 23 (12, 34) | 116 (37, 978) |
| HBV DNA  (${log}_{10} IU/ml$) * | 6.2  (3.5, 9.3) | 7.3  (3.7, 8.9) |
| HBsAg/HBsAb | 6/0 | 9/1 |
| HBeAg/HBeAb | 6/0 | 8/2 |
| HBcAb | 6 | 9 |

*Median (minimum, maximum).


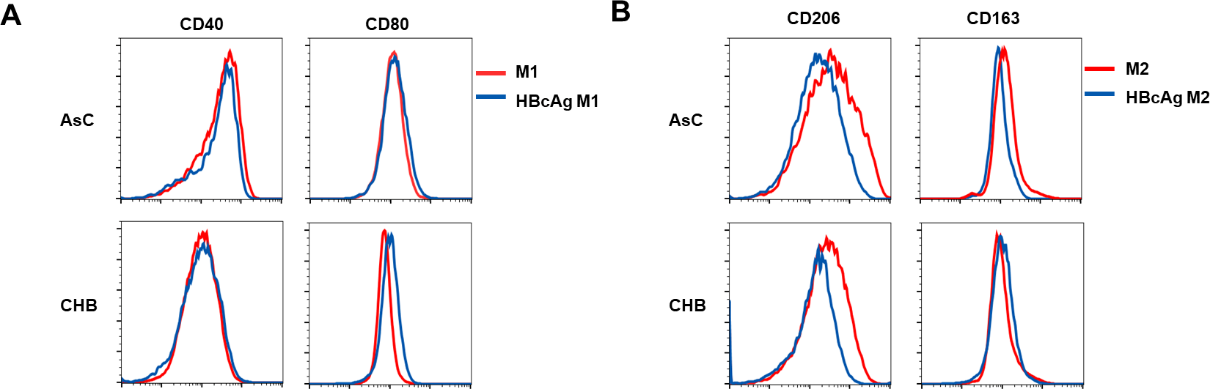


**Supplementary Figure 1. Diminished M2 phenotype from AsC or CHB patients derived macrophages.** (**A**) Representative flow cytometric graphs represented CD40 and CD80 expression levels in M1 MΦ stimulated with either HBcAg or PBS from AsC or CHB patients. (**B**) Representative flow cytometric graphs represented CD206 and CD163 expression levels in M2 MΦ stimulated with either HBcAg or PBS from AsC or CHB patients.

**
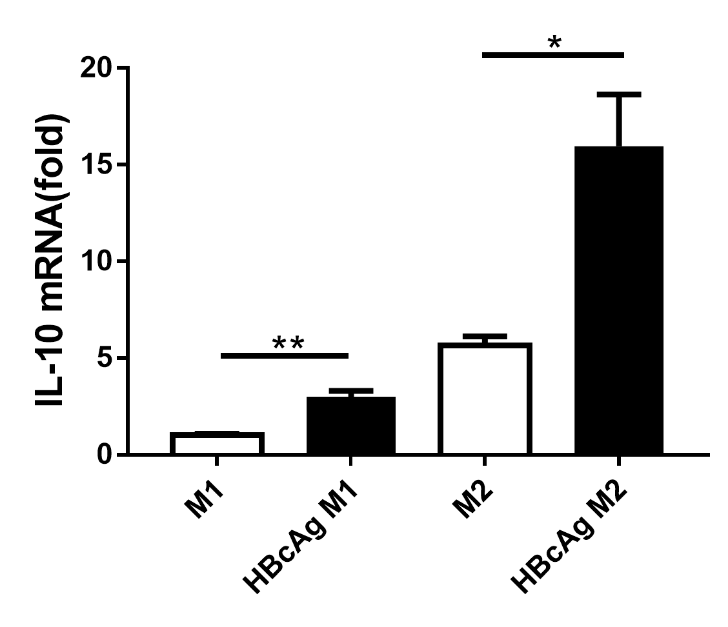
c**

**Supplementary Figure 2. HBcAg treatment promoted the expressions of IL-10 mRNA in both M1 and M2 macrophages.** IL-10 mRNA levels of M1 or M2 MΦ stimulated with either HBcAg or PBS were analyzed via RT-qPCR. Experiments were repeated at least three times. Data were analyzed using a paired Student’s *t* test. Results are shown as the mean $\pm$ SEM (n=3). **p* < 0.05, ***p* < 0.01.


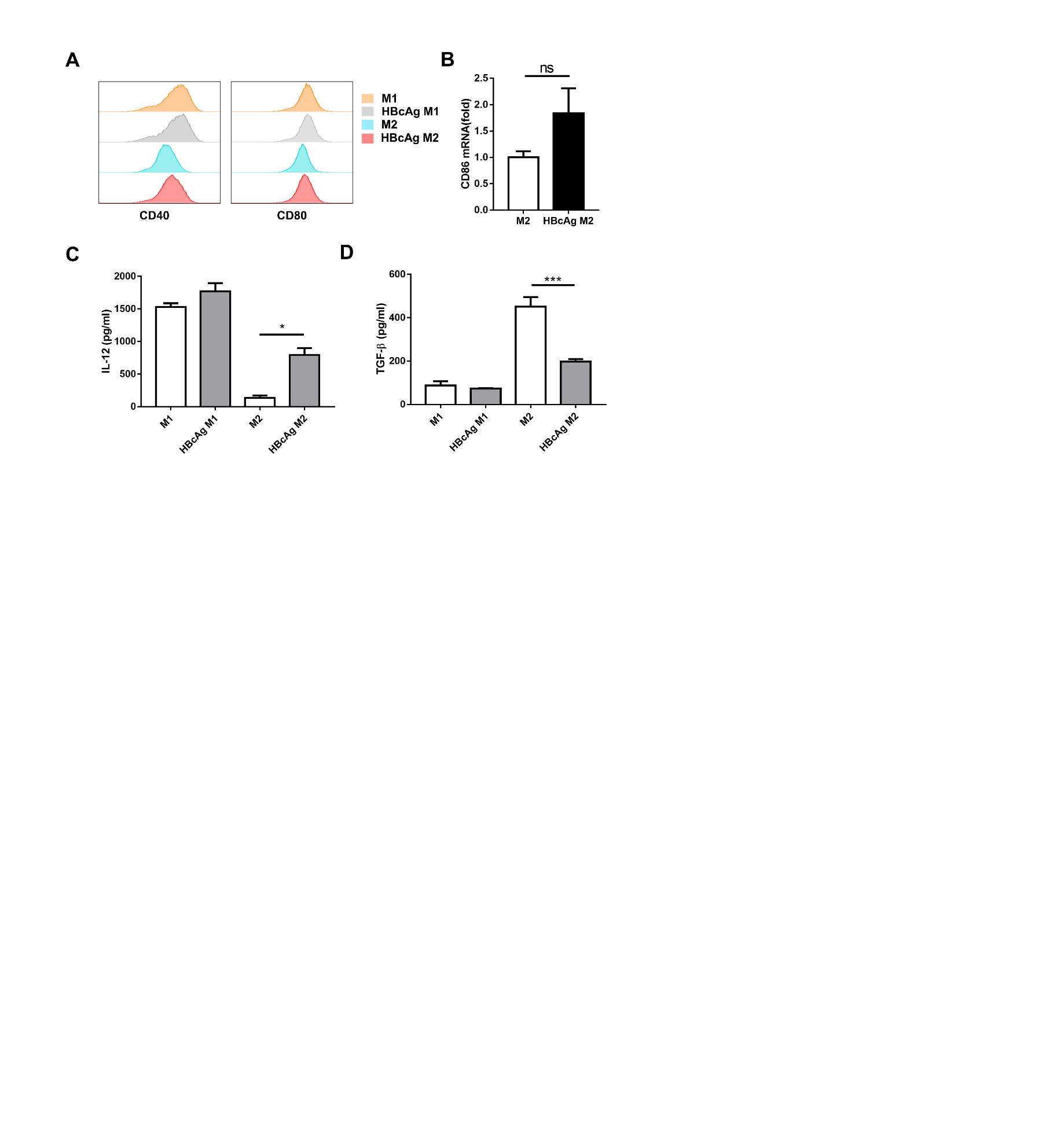


**Supplementary Figure 3. M2 macrophages treated with HBcAg did not polarize towards M2b, M2c or even M1 macrophages.** (**A**) Representative flow cytometric graphs represented CD40 and CD80 expression levels in M1 or M2 MΦ stimulated with either HBcAg or PBS. (**B**) CD86 mRNA levels of M1 or M2 MΦ stimulated with either HBcAg or PBS were analyzed via RT-qPCR. (**C-D**) Supernatants of M1 or M2 MΦ stimulated with HBcAg or PBS were collected to measure IL-12 (C) and TGF-β (D) productions by ELISA. Experiments in (A-D) were repeated at least two times. Data were analyzed using a paired Student’s *t* test. Results are shown as the mean $\pm$ SEM (B-D, n=3). **p* < 0.05, ****p* < 0.001; ns, not significant.
