# Supplementary figures and images for: Hepatitis B Core Antigen Impairs the Polarization While Promoting the Production of Inflammatory Cytokines of M2 Macrophages via the TLR2 Pathway
Source: Front Immunol. 2020 Mar 27;11:535. doi: 10.3389/fimmu.2020.00535 (PMC7118225; doi:10.3389/fimmu.2020.00535)

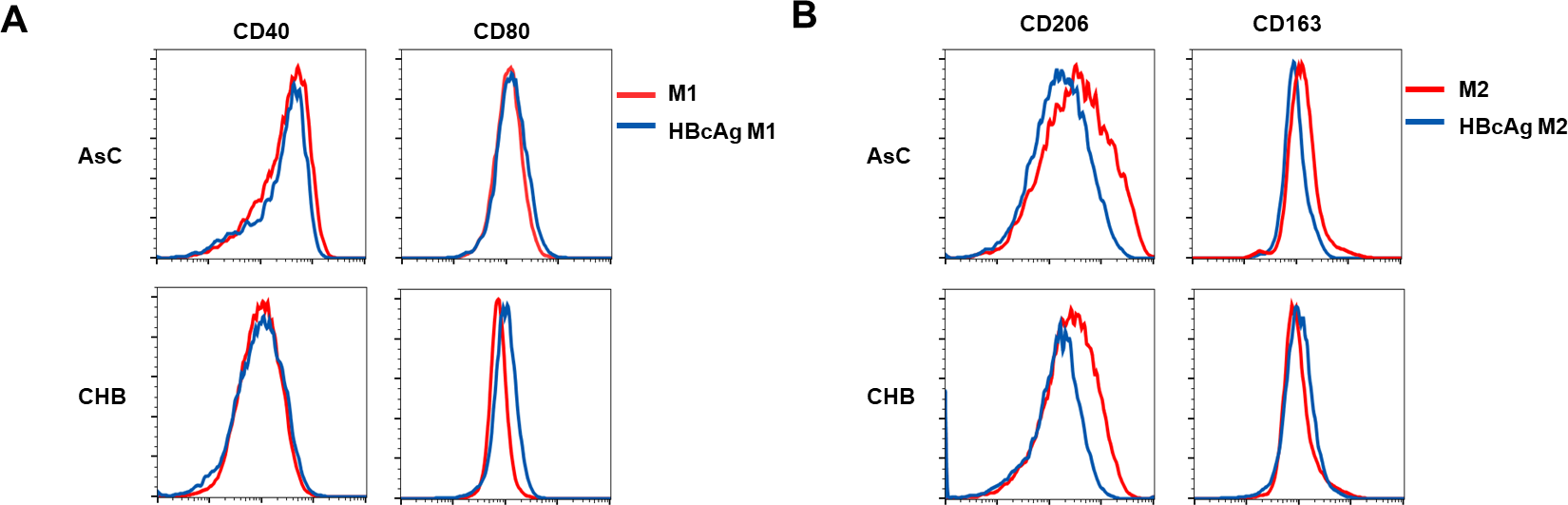

Supplement: Supplementary file 2 [file Image_1.TIF]

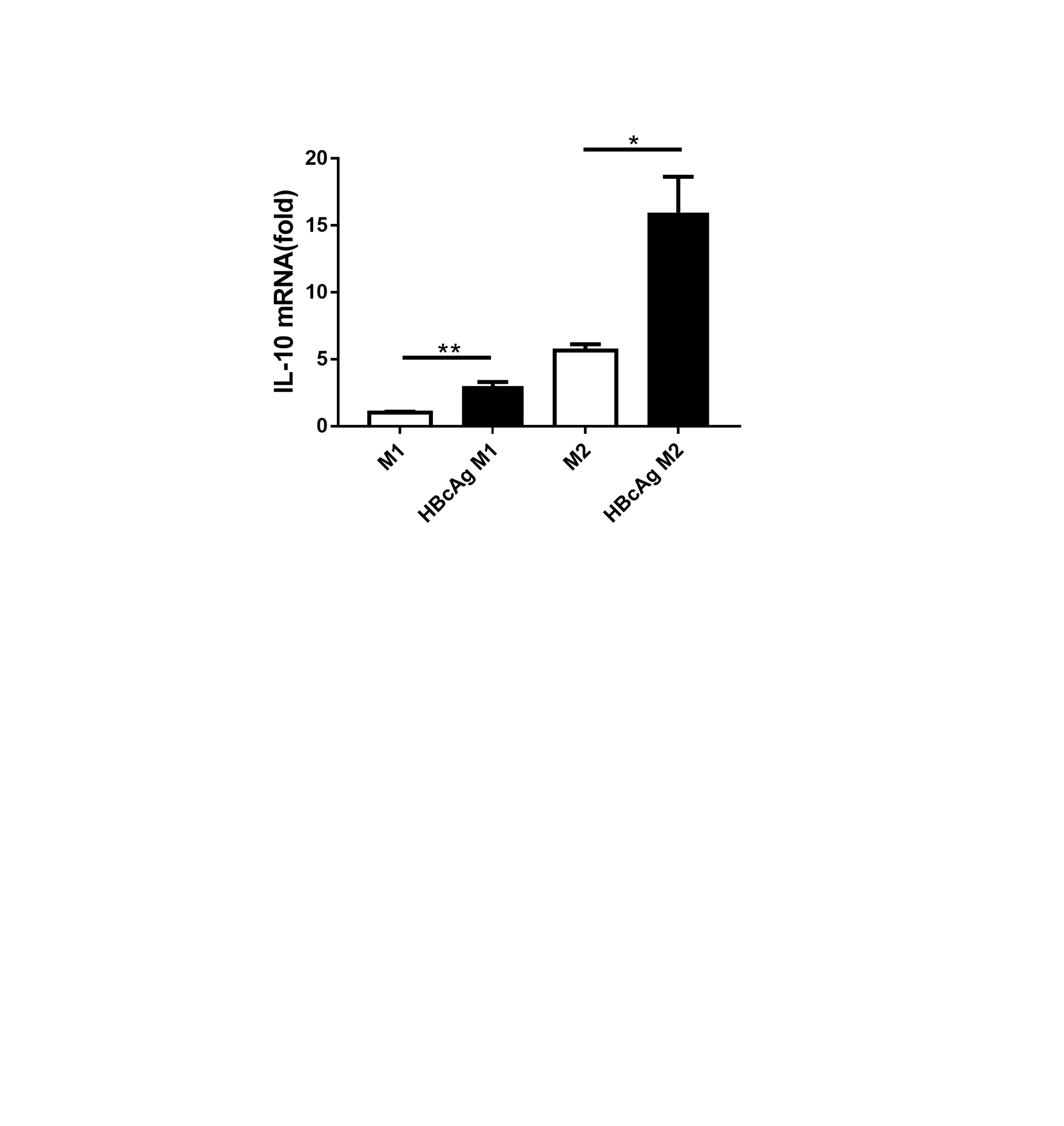

Supplement: Supplementary file 3 [file Image_2.TIF]

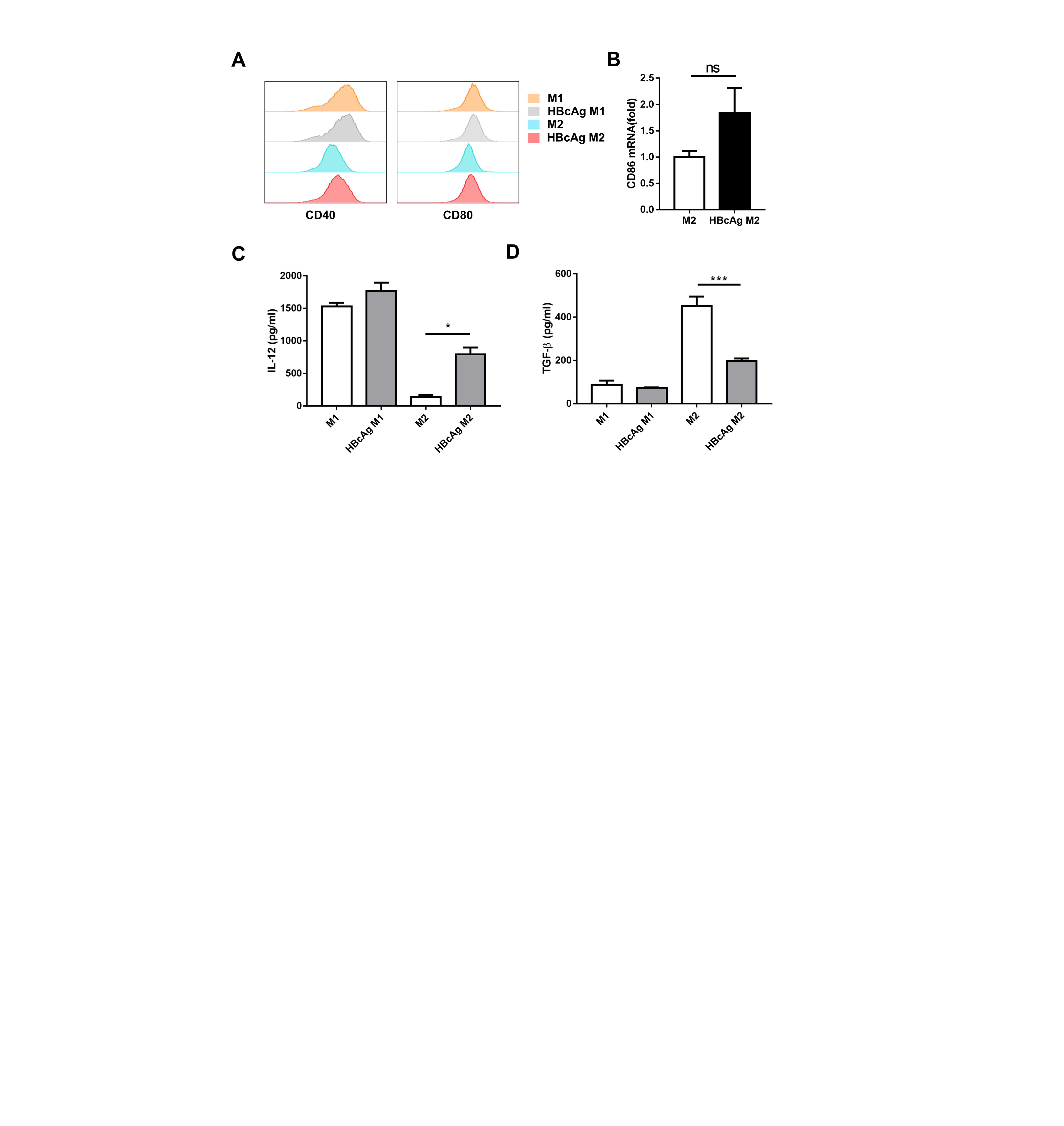

Supplement: Supplementary file 4 [file Image_3.TIF]
